# White Analytical Perspective on Lamivudine Determination: HPLC-PDA and HPLC-ELSD Method Development, Validation, and Multimetric Evaluation

**DOI:** 10.1021/acsomega.5c13207

**Published:** 2026-05-04

**Authors:** Burcu Sezgin, Göksel Arli, Ekin Aydın, Murat Soyseven

**Affiliations:** † Department of Environmental Protection Technologies, Eskişehir Vocational School, 53004 Eskişehir Osmangazi University, 26110 Eskişehir, Turkey; ‡ Department of Analytical Chemistry, Faculty of Pharmacy, 424164 Anadolu University, 26470 Eskişehir, Turkey; § Department of Pharmacy Services, Yunus Emre Vocational School of Health Services, Anadolu University, 26470 Eskişehir, Turkey; ∥ Department of Medical Services and Techniques, Yunus Emre Vocational School of Health Services, Anadolu University, 26470 Eskişehir, Turkey

## Abstract

Lamivudine (LMV) is a nucleoside reverse transcriptase inhibitor widely used in the treatment of HIV and hepatitis B infections. This study aimed to develop and validate rapid, accurate, and ecofriendly HPLC methods for the quantitative analysis of LMV in pharmaceutical formulations using photodiode array (PDA) and evaporative light scattering detector (ELSD) systems. Chromatographic separation was achieved on a C18 column (250 mm × 4.6 mm, 5 μm) using an isocratic mobile phase of ethanol–water (7:93, *v/v*) at a flow rate of 1.0 mL min^−1^. The developed methods were validated according to ICH Q2­(R2) guidelines in terms of specificity, linearity, precision, accuracy, sensitivity, and robustness. Environmental and practical sustainability were further assessed using AGREE, AGREEprep, AES, MoGAPI, SPMS, RAPI, BAGI, and CACI tools within the framework of White Analytical Chemistry (WAC). Both methods exhibited excellent linearity, with correlation coefficients (R^2^) of 0.9995 (PDA) and 0.9976 (ELSD). LOD and LOQ values were 0.12–0.40 μg mL^−1^ and 3.25–10.82 μg mL^−1^, respectively. Recovery results ranged between 98.56 and 101.42% with RSD values <2%. The AGREE, AES, and MoGAPI scores (0.72–0.69, 86–87, and 80–82%, respectively) confirmed high greenness, while BAGI (70–72.5) and CACI (73–78) values indicated strong applicability and practicality. Although the HPLC-ELSD method showed slightly lower whiteness due to its higher LOQ and energy consumption, both proposed methods demonstrated acceptable analytical performance, applicability, and environmental compatibility. The developed approaches thus provide sustainable, reliable, and cost-effective analytical platforms consistent with the principles of WAC.

## Graphical abstract

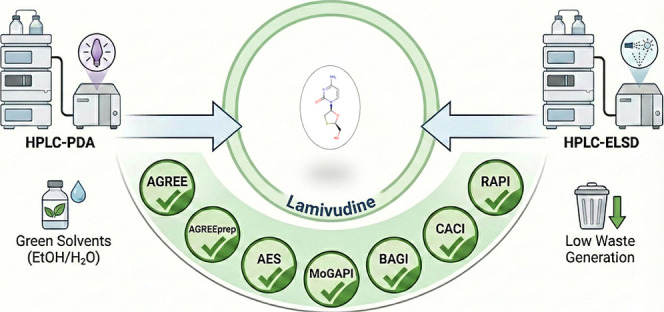

## 1 Introduction

The Human Immunodeficiency Virus (HIV) is one of the most significant global public health challenges of our time. As a retrovirus that compromises the human immune system, it leads to Acquired Immunodeficiency Syndrome (AIDS). Effective antiretroviral drugs (ARVs) are of vital importance in the management and treatment of this devastating disease.^1–4^ Within the standard therapeutic approach aimed at preventing viral transmission and significantly reducing HIV-associated morbidity and mortality rates, nucleoside and nucleotide structural analogs known as nucleoside reverse transcription inhibitors (NRTIs) play a crucial role. NRTIs competitively inhibit viral reverse transcriptase, thereby halting viral DNA synthesis and suppressing viral replication. Among this critical class of drugs, Lamivudine (LMV), a synthetic nucleoside analog exhibiting activity against HIV-1, HIV-2, and the hepatitis B virus, serves as a representative example.^1,5–7^

Chemically known as (2R-cis)-4-amino-1-[2-(hydroxymethyl)-1,3-oxathiolan-5-yl]-2­(1H)-pyrimidinone, LMV is particularly notable for exhibiting low cellular cytotoxicity.^8–10^ To exert its therapeutic effect, LMV undergoes intracellular phosphorylation to form its active metabolite, LMV triphosphate. This active metabolite competitively inhibits viral reverse transcriptase, thereby preventing HIV replication and terminating the elongation of the viral DNA chain.^11,12^ When administered orally, LMV is rapidly absorbed and exhibits high bioavailability, exceeding 80% in adults.^10,13^ Commercial formulations include 100 or 150 mg capsules/tablets and 10 mg mL^−1^ syrups for pediatric use.^9,12^ The molecular structure of LMV is shown in Figure 1.^14^

**Figure 1:**
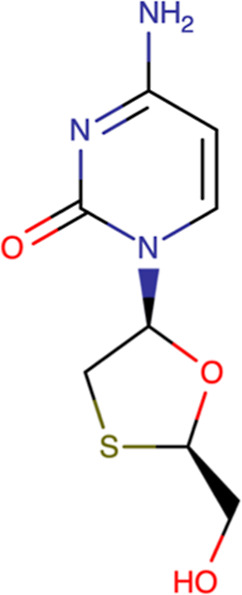
Molecular structure of LMV.

Various analytical approaches have been reported in the literature for the determination of LMV in different matrices, among which Liquid Chromatography (LC) stands out owing to its exceptional sensitivity, precision, and efficiency in resolving complex mixtures.^15,16^ The reported techniques for the determination of LMV are High-Performance Liquid Chromatography (HPLC),^1,9,10,17^ Ultra-Performance Liquid Chromatography (UPLC),^5,18^ Mass Spectrometry (MS/MS)-based LC methods,^2,7,15^ spectrophotometry,^3,4,8,11,19^ voltammetric techniques,^12^ Raman spectroscopy,^20^ and Micellar Electrokinetic Chromatography (MEKC).^21^

Over the past few years, Green Analytical Chemistry (GAC) and White Analytical Chemistry (WAC) have emerged as key paradigms in advancing sustainable analytical science. GAC emphasizes reducing the ecological footprint of analytical processes, whereas WAC extends this approach by integrating environmental, economic, and practical aspects with analytical quality. The combination of these two concepts fosters the creation of analytical methods that are environmentally conscious, reliable, cost-effective, and applicable to real-world scenarios.^22–24^ The trend toward comprehensive sustainability evaluation in analytical chemistry has accelerated, with multimetric assessment approaches becoming increasingly popular. Rather than relying on a single greenness or performance indicator, researchers employ a combination of complementary tools to obtain a more balanced and realistic picture of a method’s overall sustainability profile. This multimetric strategy not only captures the environmental dimension but also considers analytical efficiency, operational simplicity, cost, and safety, aligning perfectly with the holistic philosophy of modern GAC and WAC. As a result, multimetric assessment has evolved into a standard practice in contemporary method development, supporting both ecological responsibility and analytical excellence.^22,25,26^

Despite the availability of several chromatographic methods for LMV determination, limited attention has been given to decision-oriented comparisons between detection techniques within the framework of WAC. The research aims to develop and validate novel, ecoconscious analytical methods for the quantification of LMV in pharmaceutical formulations. The objective is to establish rapid, simple, accurate, precise, robust, cost-effective, and environmentally sustainable chromatographic methods suitable for routine quality control and regulatory applications. In this context, green, sensitive, and reliable HPLC-PDA and HPLC-ELSD methods were developed and validated. Moreover, the environmental and practical performance of the proposed methods was comprehensively examined through multiple greenness and whiteness evaluation metrics. This integrated assessment framework not only demonstrates the analytical efficiency of the developed methods but also emphasizes their alignment with the principles of sustainable and responsible analytical chemistry.

A review of the literature reveals that most existing studies on LMV determination have primarily focused on chromatographic optimization or limited green assessment, while research integrating broader sustainability criteria remains scarce. Although numerous HPLC-UV and HPLC-PDA methods have been reported, comparative evaluations of detectors in terms of eco-efficiency and whiteness (holistic sustainability) are notably lacking. Furthermore, studies combining analytical validation with multimetric greenness and whiteness assessments are extremely limited. Notably, no study to date has reported the determination of LMV using an HPLC-ELSD system. In this respect, the present work not only introduces the first HPLC-ELSD method for LMV analysis but also represents the first comprehensive WAC approach comparing HPLC-PDA and HPLC-ELSD techniques in terms of analytical performance, environmental impact, and economic sustainability. Thus, this study fills a significant gap in the literature by offering both scientific novelty and a holistic sustainability perspective, serving as a model for future environmentally conscious pharmaceutical analyses.

## 2 Experimental Section

### 2.1 Chemicals and Materials

The LMV standard was supplied from Sigma-Aldrich (Germany) as > 98% purity. HPLC-grade ethanol (EtOH) was purchased from Merck (Germany). Ultrapure water system was used for all standard and sample solution preparation, with conductivity of 0.055 μS/cm supplied from Stakpure (Germany). A 250 mm × 4.6 mm i.d., 5 μm particle size, 110 Å pore size HPLC C18 SVEA column (Nanologica, Sweden) was used for both HPLC-PDA and HPLC-ELSD analyses. All working standards and sample solutions were filtered through nonsterile PVDF syringe filters (0.22 μm pore size, Sigma-Aldrich, Germany) prior to injection into the HPLC system.

### 2.2 Apparatus

An HPLC system (Shimadzu Nexera-i LC2040C 3D Tokyo, Japan) consisting of a binary pump, a degasser, an autosampler, a thermostatic column compartment combined with a PDA (Shimadzu Tokyo, Japan), and an ELSD (Shimadzu ELSD-LT-II Model Tokyo, Japan) were used for all the analyses. All the working standards and samples were sonicated with a LabCompanion CS-10 ultrasonic cleaner (Korea) and vortexed with a Heidolph Vortex (Germany). An Ohaus brand analytical balance was used at every weighing step of the standard and sample preparation (United States). pH measurements were conducted with a SevenMulti pH meter supplied by Mettler-Toledo (Switzerland). Chromatographic data were processed via LabSolution software (Shimadzu, Japan) and Microsoft Excel, version 16.97.2 (USA).

### 2.3 Chromatographic Conditions

An isocratic elution method was employed during all HPLC-PDA and HPLC-ELSD analyses, with the mobile phase composed of water:EtOH (93:7, *v/v*). Separations were performed using a C18 column (5 μm particle size, 250 mm × 4.6 mm i.d., 110 Å pore size). The mobile-phase flow rate was adjusted as 1.0 mL min^−1^ to the HPLC-PDA and HPLC-ELSD systems. For HPLC-PDA analysis, the detection wavelength was set at 270 nm, corresponding to the maximum absorbance of LMV. The spectra were recorded within the wavelength range of 190–800 nm. In the case of HPLC-ELSD, the operating parameters were adjusted as follows: nitrogen gas pressure 350 kPa; gain 7; filter 10; and evaporator temperature 50 °C. The column temperature was maintained at 40 °C, with an injection volume of 10 μL. Each run was completed within 10 min in both PDA and ELSD.

### 2.4 Preparation of the Standard Stock Solutions, Sample Solution, and Mobile Phases

#### 2.4.1 Standard Stock Solution of LMV (1000 μg mL^−1^ in Water)

A standard stock solution of LMV was prepared by dissolving 25 mg of LMV in 25 mL volumetric flasks, using HPLC-grade water as the solvent. All solutions were stored at 4 °C in the refrigerator during experiments. Each standard was prepared in HPLC-grade water for LMV, and all dilutions were made by water.

#### 2.4.2 Sample Solutions

For the preparation of LMV sample solutions, ten tablets from commercial brand were accurately weighed, finely powdered, and homogenized. The mean tablet weights were determined to be 0.229 g. A precisely measured quantity equivalent to 100 mg of LMV was then transferred to a 100 mL volumetric flask, along with water. The mixture was vigorously shaken and subjected to sonication for 15 min to ensure complete dissolution and homogenization of the active substance. The volume was completed with water, and the resulting solutions were filtered through 0.22 μm PVDF syringe filters to remove particulate matter. Subsequent dilutions were prepared by adding water. The clear filtrates were then transferred into HPLC vials and stored until analysis by HPLC. The analysis procedure followed for each method included linearity assessment, as well as the construction of calibration curves. Based on this analysis, the concentration levels for LMV present in the samples were determined.

#### 2.4.3 Mobile Phase

The mobile phase employed for chromatographic analysis consisted of an aqueous–organic mixture containing 93% (v/v) HPLC-grade water and 7% (v/v) EtOH. Both solvents were of HPLC grade to ensure high purity and to minimize background noise or interference during analysis. To ensure complete miscibility of the solvents and removal of any dissolved air bubbles, the solution was placed in an ultrasonic bath (sonicator) for 15 min. This sonication process enhanced homogenization of the binary solvent system and facilitated degassing. Following sonication, the mobile phase was filtered through nonsterile regenerated cellulose membrane filters (50 mm id, 0.2 μm pore size, from Whatman, Germany) using a vacuum filtration system. This step was carried out to remove any particulate matter that could otherwise clog the HPLC column or interfere with the detector performance.

### 2.5 Validation

To ensure the reliability and quality of the analytical procedure, the optimized HPLC-PDA and HPLC-ELSD methods were validated in accordance with the International Conference on Harmonisation (ICH) guidelines Q2­(R2).^27^ The validation parameters include linearity, limit of detection (LOD), limit of quantification (LOQ), accuracy, precision, specificity, and robustness. The system suitability tests (SSTs) were thoroughly examined with respect to key chromatographic parameters such as tailing factor (T), capacity factor (k’), and theoretical plate count (N). The obtained results were processed using Shimadzu LabSolution software. All experiments were made in triplicate (*n* = 3). All statistical analyses were calculated using Microsoft Excel software, version 16.97.2 (USA).

### 2.6 Whiteness Assessment

The reliability and applicability of the proposed HPLC-PDA and HPLC-ELSD methods were systematically examined through multiple performance indices, including the Red Analytical Performance Index (RAPI),^28^ the Blue Applicability Grade Index (BAGI),^29^ and the Click Analytical Chemistry Index (CACI).^30^ These evaluations provided an integrated assessment of the analytical efficiency and practical applicability of the methods. In addition, the environmental sustainability of the developed approaches was thoroughly investigated by using both qualitative and quantitative greenness metrics. Specifically, the Green Solvent Selection Tool (GSST),^31^ Analytical Eco-Scale (AES),^32^ the Analytical Greenness Metric (AGREE),^33^ the Analytical Greenness Metric for Sample Preparation (AGREEprep),^34^ the Sample Preparation Metric of Sustainability (SPMS),^35^ and the Modified Green Analytical Procedure Index (MoGAPI)^36^ were employed to comprehensively evaluate the ecological profile of the methods.

RAPI is an assessment tool developed to quantitatively evaluate the analytical performance of methods, particularly in relation to validation parameters. Rooted in the principles of WAC and conceptually aligned with the red dimension of the RGB model, RAPI systematically examines ten key validation attributes, including trueness, repeatability, selectivity, and LOQ. Each parameter is independently scored on a 0–10 scale, yielding an overall performance index ranging from 0 to 100. The outcomes are graphically depicted as a red-shaded star-shaped diagram, where all parameters are assigned equal weighting. By focusing on validation quality rather than environmental or practical aspects, RAPI provides a complementary perspective to greenness and whiteness evaluation tools, thereby facilitating a more holistic appraisal of analytical methods in terms of reliability and applicability.^28^

BAGI is a performance metric developed to assess the practical applicability and operational efficiency of analytical methods under routine laboratory conditions. Within the framework of the WAC concept, BAGI complements eco-efficiency-oriented green metrics by focusing on parameters related to real-world usability, throughput, and resource economy. The index evaluates analytical methods across ten operational criteria, including the nature of the analytical approach, number of analytes determined per run, instrumental complexity, extent and duration of sample preparation, degree of automation, and accessibility of reagents and equipment. Each parameter is assigned a numerical score, and the overall BAGI value is visually represented by a blue-shaded asteroid-shaped diagram, where higher scores correspond to greater methodological practicality. Based on the generally accepted interpretive scale, a score ≥60 denotes satisfactory operational applicability.^29^

CACI is a multidimensional metric developed to assess analytical methods with respect to their practicality, operational efficiency, and overall usability. Designed to encourage the adoption of cost-effective, rapid, and straightforward analytical procedures, CACI emphasizes minimizing resource consumption while maintaining analytical reliability. The scoring system is based on a point-based evaluation encompassing several performance-related criteria, including sample size requirements, sample preparation complexity, cost per analysis, degree of automation, portability of instrumentation, analytical sensitivity, total analysis time, and applicability to diverse sample matrices.^30^

AES is a semiquantitative tool designed to evaluate the environmental performance of analytical procedures by assigning penalty points to parameters that diverge from the principles of an “ideal green analysis.” The system is based on a reference score of 100, which represents a completely green analytical process. Penalty points (PPs) are allocated across several critical aspects of the method, including reagent consumption, energy demand, occupational safety risks, waste generation and management, and the number of procedural steps involved. The overall AES score is obtained by deducting the total PPs from 100, with higher scores corresponding to greener methodologies. According to the interpretative scale, methods scoring above 75 are classified as “excellent green,” those above 50 as “acceptable green,” and those below 50 as “inadequate”.^32^

The AGREE metric offers a systematic approach to assessing the environmental sustainability of analytical methods based on the twelve principles of GAC. Each principle is scored from 0 to 1, and the results are visually displayed in a clock-shaped diagram using color gradients from red to green, allowing rapid interpretation of overall and individual performance.^33^

AGREEprep, a complementary tool to AGREE, specifically evaluates the greenness of sample preparation steps, providing a more detailed insight into a method’s environmental impact. It assesses ten criteria, including solvent and reagent use, waste generation, energy demand, automation level, and operator safety, with higher weights assigned to environmentally critical factors such as solvent consumption and safety.^34^

SPMS tool is specifically developed to evaluate the environmental sustainability of sample preparation processes. Unlike general greenness metrics, it focuses solely on the sample preparation stage, allowing for a more accurate comparison and optimization of the extraction techniques. The SPMS framework assesses nine core parameters organized into four principal categories: sample characteristics, extractant properties, procedural aspects, and energy/waste outputs.^35^

The MoGAPI metric evaluates analytical methods based on 17 criteria encompassing sample preparation, solvent and reagent consumption, instrumentation, waste generation, and quantification approach. Each parameter is rated on a 3-point scale (1–3), and the overall greenness score is expressed as a percentage by dividing the total obtained score by the maximum possible score.^36^

## 3 Results and Discussion

### 3.1 Method Development

The primary objective of this study was to develop rapid, simple, reliable, sensitive, and environmentally friendly HPLC-PDA and HPLC-ELSD methods for the quantification of LMV in pharmaceutical formulations. To minimize environmental impact, greener solvent systems were employed by replacing traditionally used toxic organic solvents such as methanol (MeOH) and acetonitrile (ACN) with EtOH and water. Notably, EtOH is recognized as a Generally Recognized As Safe (GRAS) solvent by the US Food and Drug Administration (FDA), further strengthening the green chemistry compliance of the proposed method.

Preliminary trials indicated that the use of a C18 column with EtOH/water mixtures as the mobile phase provided significant advantages in terms of solubility for polar and ionizable analytes while simultaneously ensuring sustainability and reduced toxicity. Composite G-score evaluations yielded values of 7.3 for water and 6.6 for EtOH (Figure 2), confirming their environmental preferability and suitability for green method development. Accordingly, mobile phases were prepared by varying the EtOH-to-water ratios, thereby optimizing the chromatographic performance while adhering to the principles of GAC.

**Figure 2:**
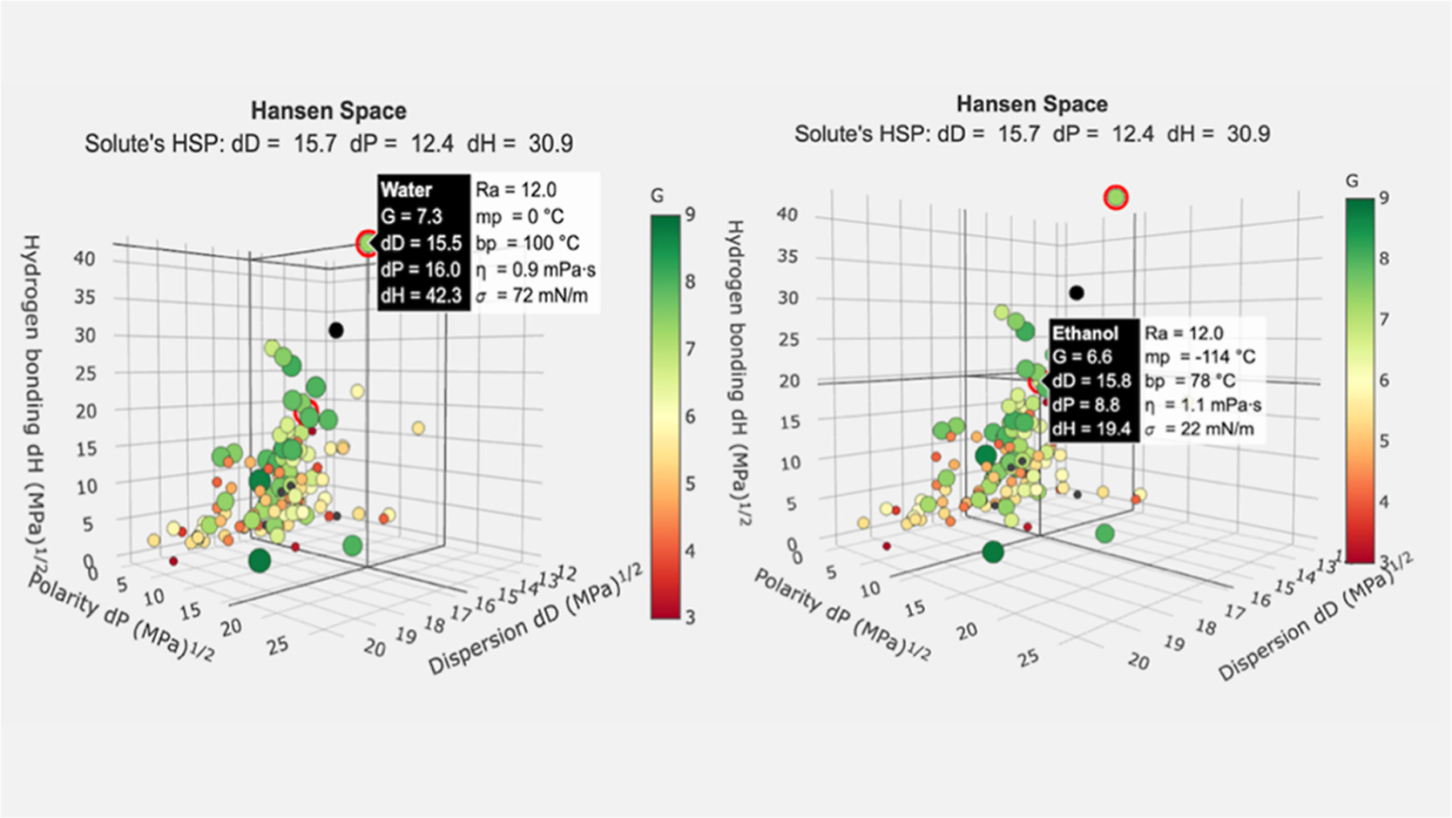
Examined G scores of the mobile-phase solvents by GSST.

All HPLC analyses were performed under isocratic elution conditions. To obtain optimal chromatographic resolution and peak symmetry, various EtOH–water compositions were systematically tested as the mobile phase. The objective of the mobile phase optimization was not to establish conditions suitable for a single detector but rather to identify a balanced and reliable composition that simultaneously satisfies the system suitability criteria for both PDA and ELSD detection. To achieve this, a broader range of ethanol concentrations was systematically evaluated. The decision-making process was based on the joint assessment of key system suitability parameters, particularly the tailing factor (T) and capacity factor (k’), for both detectors. While minimizing organic solvent consumption was considered within the optimization framework, chromatographic performance criteria remained the primary determinant. Special attention was given to conditions approaching critical acceptance limits. At 6% EtOH, the tailing factor for ELSD exceeded the predefined acceptance criterion (*T* > 2.0), indicating inadequate peak symmetry. Conversely, at 8% EtOH, the capacity factor for ELSD decreased below the recommended lower limit (k’ < 1), suggesting insufficient retention robustness. The data corresponding to these boundary conditions, which were decisive in the selection process, are presented in Table 1. The 7% EtOH composition was ultimately selected, as it simultaneously met the acceptance criteria for both capacity factor and peak symmetry in both detection systems without approaching critical thresholds, thereby representing the most balanced and robust condition.

**Table 1:** Effect of EtOH Concentration on System Suitability Parameters

parameter	acceptance criteria	detector	EtOH %		
			6	7	8
retention time (min)	–	PDA	5.63	5.50	5.40
		ELSD	5.60	5.49	5.41
tailing factor (T)	≤2.0	PDA	1.41	1.39	1.36
		ELSD	2.01	1.93	1.91
capacity factor (k’)	1 ≤ k’ ≤ 10	PDA	1.19	1.14	1.10
		ELSD	1.06	1.02	0.99

Additionally, extensive optimization studies were carried out for both the PDA and ELSD detection systems. For the PDA detector, different wavelengths were tested to maximize sensitivity and selectivity, while for the ELSD system, parameters such as drift tube temperature, gain, filter settings, and nitrogen gas pressure were varied to achieve optimal signal stability and reproducibility. The final optimized chromatographic parameters for both HPLC-PDA and HPLC-ELSD methods are summarized in Section 2.3. The chromatograms of LMV monitored by the HPLC-PDA and HPLC-ELSD methods are given in Figure 3.

**Figure 3:**
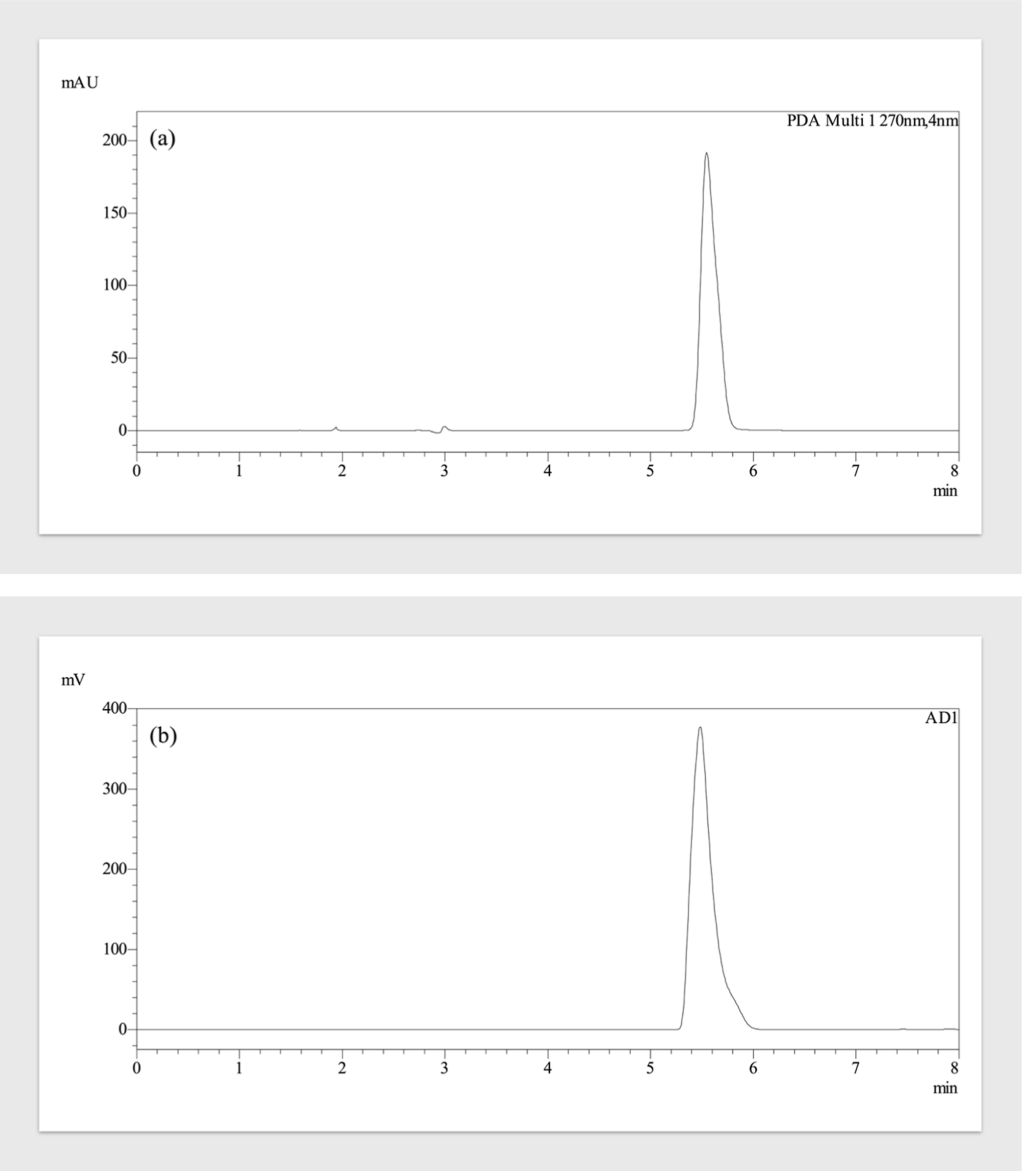
Chromatograms of the LMV standard solutions (a) HPLC-PDA (100 μg mL^−1^) and (b) HPLC-ELSD (1000 μg mL^−1^) at optimum conditions.

### 3.2 Validation

#### 3.2.1 SST Parameters

SST parameters (T, k’, and N) represent a critical component of the validation process, as they provide a direct measure of the efficiency and reliability of chromatographic separation in HPLC analyses. In this study, the SST results for the developed HPLC-PDA and HPLC-ELSD methods were obtained by using Shimadzu LabSolutions software, which integrated the chromatographic data. The calculated parameters demonstrated that the system performance met the required criteria, confirming the robustness of the developed methods. The detailed SST results are presented in Table 2.

**Table 2:** SST Results of the HPLC-PDA and HPLC-ELSD Methods (*n* = 6)^a^

parameter	acceptance criteria	HPLC-PDA	HPLC-ELSD
retention time (t_R_, min)	–	5.50	5.49
RSD of t_R_ (%)	≤1.0	0.05	0.06
RSD of peak area (%)	≤2.0	0.09	0.99
theoretical plates (N)	≥2000	6439	4306
tailing factor (T)	≤2.0	1.39	1.93
capacity factor (k’)	1 ≤ k’ ≤ 10	1.14	1.02

a RSD: Relative standard deviation.

SST parameters were well within the acceptance limits. The low RSD of retention time (≤0.06%) and peak area (≤1%) further confirmed chromatographic stability. These values indicate a well-optimized stationary/mobile phase interaction and efficient mass transfer across the column, ensuring sharp and symmetrical peaks.

#### 3.2.2 Specificity and Selectivity

Specificity is generally defined as the ability of an analytical method to unambiguously measure the target analyte in the presence of potential interferences, thereby ensuring accurate identification and quantification of the analyte of interest. Selectivity, on the other hand, is a relative term that describes the extent to which an analyte can be reliably measured in complex mixtures or matrices without interference from other components exhibiting similar chemical behavior. The selectivity of the developed HPLC-PDA and HPLC-ELSD methods was evaluated based on their ability to effectively separate LMV from potential interfering substances. Examination of the obtained chromatograms revealed that the LMV peak was well-resolved, with no evidence of coeluting or interfering peaks. The calculated selectivity factor (α > 1.00) met the recommended criterion, confirming that the methods provide sufficient selectivity for the reliable quantification of LMV in pharmaceutical matrices. The PDA detection additionally allowed spectral purity confirmation through peak purity analysis, strengthening the method reliability. In addition, potential interference from common pharmaceutical excipients was evaluated by analyzing sample solutions prepared from commercial tablets. No additional peaks were observed at the retention time of LMV, confirming the absence of matrix interference. Furthermore, at the selected PDA detection wavelength (270 nm), no coeluting peaks were detected, and peak purity analysis confirmed the spectral homogeneity of LMV, indicating that no other components interfered with the detection.

#### 3.2.3 Linearity, LOD, and LOQ

Linearity refers to the ability of an analytical method to produce results that are directly proportional to the concentration (or amount) of the analyte within a defined range. It is a fundamental validation parameter, as it ensures that the method can generate accurate and precise results across the reportable range.^27^

For the developed HPLC-PDA and HPLC-ELSD methods, linearity was evaluated over concentration ranges of 2–200 μg mL^−1^ and 30–1200 μg mL^−1^, respectively. Unlike UV-based detectors, ELSD does not provide a direct linear response and therefore does not obey Beer–Lambert’s law. Instead, the detector response follows a nonlinear empirical function, and logarithmic transformation of both peak area and concentration is routinely applied to achieve linear calibration behavior.^25,26^ Calibration curves were constructed by plotting chromatographic peak areas against analyte concentrations for the HPLC-PDA method, while in the case of HPLC-ELSD, calibration was performed using logarithmically transformed data (*logC*–*logArea*), in accordance with established practices for ELSD-based quantification.

The use of different concentration ranges for the HPLC-PDA and HPLC-ELSD methods stems from the fundamental differences in how these detectors respond to and their sensitivity levels. PDA detection, based on UV absorbance, is generally more sensitive, allowing for the measurement of lower analyte concentrations. In contrast, ELSD is less sensitive by nature and requires higher analyte concentrations to produce a sufficient signal. For this reason, separate calibration ranges were chosen for each detector to ensure an accurate and reliable quantification.

The linear regression equations indicate excellent linearity across the tested ranges. Statistical evaluation was performed using the least-squares regression approach, further confirming the robustness of the calibration models. The results collectively demonstrate that the developed HPLC-PDA and HPLC-ELSD methods provide highly linear responses within the studied concentration ranges, thereby validating their suitability for the reliable quantitative analysis of LMV.

The LOD was calculated using a signal-to-noise (S/N) ratio of 3:1. Determination of the LOD is particularly critical for qualitative methods and impurity profiling, where the presence of low-level components must be confirmed with confidence. The LOQ was calculated based on a signal-to-noise (S/N) ratio of 10:1, ensuring reliable quantification at low concentration levels.^27^ The results are summarized in Table 3.

**Table 3:** Linearity, LOD, and LOQ of the Developed Methods

parameter	HPLC-PDA	HPLC-ELSD
range (μg mL^−1^)	2–200	30–1200
regression equation	*y* = 17966x + 52178	*y* = 1.6916x + 1.7059
*R* ^*2*^	0.9995	0.9976
LOD (μg mL^−1^)	0.12	3.25
LOQ (μg mL^−1^)	0.40	10.82

The calibration curves showed outstanding linearity over wide concentration ranges (2–200 μg mL^−1^ for PDA and 30–1200 μg mL^−1^ for ELSD), with correlation coefficients (*R* ^*2*^) of 0.9995 and 0.9976, respectively. The broader linear range of the ELSD method indicates its suitability for higher-concentration analyses, whereas the PDA method offers superior sensitivity. In contrast, the ELSD limits (3.25 and 10.82 μg mL^−1^) were approximately an order of magnitude higher, consistent with the inherent lower sensitivity of scattering-based detection. These results demonstrate that the PDA detector is preferable for trace quantification, while the ELSD system provides a viable alternative when the UV transparency is limited.

It should be noted that the higher LOQ observed for the HPLC-ELSD method (10.82 μg mL^−1^) is primarily attributed to the intrinsic detection mechanism of ELSD, which relies on light scattering rather than direct absorbance. Despite systematic optimization of ELSD operating parameters, including nebulizer gas pressure, evaporator temperature, gain, and filter settings, the sensitivity of ELSD remains inherently lower than that of PDA, particularly for highly polar and low-molecular-weight compounds such as LMV. Although further sensitivity enhancement could theoretically be achieved through additional strategies such as sample preconcentration or solvent exchange, these approaches were deliberately avoided in the present study to maintain a simple, rapid, and environmentally compatible analytical workflow in accordance with the principles of WAC. Importantly, the achieved LOQ is fully adequate for pharmaceutical quality control applications, where LMV is present at relatively high concentration levels. Consequently, although HPLC-PDA is more suitable for trace-level analysis, the HPLC-ELSD method offers a complementary, green, and practically applicable alternative within the holistic WAC framework.

#### 3.2.4 Accuracy and Precision

In this study, the trueness of the method was quantitatively assessed by a spiking recovery test. Pre-analyzed pharmaceutical samples were fortified with known concentrations of the LMV standard solution at three levels for HPLC-PDA and HPLC-ELSD methods (10, 80, 150 μg mL^−1^ and 60, 200, 800 μg mL^−1^, respectively) corresponding to the lower, middle, and upper portions of the calibration range. Each spiking level was prepared and analyzed in triplicate (*n* = 3). The recovery (%) was calculated as the ratio of the measured concentration to the added concentration expressed as a percentage. Detailed results are summarized in Table 4.

**Table 4:** Accuracy and Precision Results of the Developed Method

	test value (μg mL^−1^)	accuracy	precision (^b^RSD %)	
method		(^a^recovery %)	repeatability	intermediate precision
	10	98.74	0.88	0.84
**HPLC-PDA**	80	101.42	0.76	0.61
	150	99.84	0.45	0.56
	60	101.01	0.06	0.47
**HPLC-ELSD**	200	101.06	0.02	0.69
	800	98.56	0.07	0.49

a Recovery % = [the measured value/(the existed + the spiked value)] × 100.

b RSD % = [SD/mean] × 100.

In this work, precision was assessed at two hierarchical levels: repeatability (*intraday* precision) and intermediate precision (*interday* precision), in accordance with ICH Q2­(R2) guidelines.^27^ Repeatability was evaluated by analyzing three concentration levels for HPLC-PDA and HPLC-ELSD methods (10, 80, 150 μg mL^−1^ and 60, 200, and 800 μg mL^−1^, respectively) in triplicate within the same analytical run (*n* = 9) under identical operating parameters. Intermediate precision was determined by repeating the same experiment on different days and by different analysts using the same chromatographic system, reagents, and calibration solutions. The relative standard deviation (RSD, %) was calculated for each concentration level to quantify variability. All RSD values were within the acceptance limits (<2%), demonstrating that the developed method ensures high precision and robustness under both constant and variable analytical conditions. The obtained precision data are presented in Table 4.

Accuracy studies revealed recovery values within 98–102% at three spiking levels, confirming the method trueness and the absence of matrix interference. The calculated RSD values (<1%) at each level indicate minimal random error and robust reproducibility. The intraday (repeatability) and interday (intermediate precision) evaluations yielded RSD values below 2% for both detectors, underscoring their high stability over time and across analysts. The PDA system exhibited slightly lower variability (RSD ≤0.8%) compared to ELSD (RSD ≤0.9%), which may be attributed to the improved signal-to-noise ratio and more stable baseline of photometric detection.

#### 3.2.5 Robustness

The robustness was systematically evaluated to assess the reliability of the developed HPLC-PDA and HPLC-ELSD methods under minor operational deviations. Controlled changes were introduced in key chromatographic parameters, including flow rate (±0.1 mL min^−1^), organic phase ratio (±2%), and column temperature (±2 °C). Solutions containing LMV at nominal concentrations of 20 μg mL^−1^ and 200 μg mL^−1^ were used for robustness testing with the HPLC-PDA and HPLC-ELSD systems, respectively. Each condition was tested through triplicate injections to ensure statistical reliability. %RSDs of the peak areas were calculated to assess the influence of each parameter variation on the quantitative response. The summarized results are listed in Table 5.

**Table 5:** Robustness Results of the Developed Methods (*n* = 3)^a^

method	parameter	tested value	peak area (mAU·s)	RSD (%)
**HPLC-PDA**	**flow rate**(mL min^−1^)	1.0	424083	0.98
		1.1	425594	0.95
		0.9	424456	1.02
	**organic modifier**(EtOH %)	7.0	424083	0.96
		7.1	425452	0.94
		6.9	425556	1.04
	**column temperature**(°C)	40	424083	1.15
		42	425302	1.22
		38	424856	0.97
**HPLC-ELSD**	**flow rate**(mL min^−1^)	1.0	481667	0.92
		1.1	482658	0.98
		0.9	482084	1.03
	**organic modifier**(EtOH %)	7.0	481667	1.10
		7.1	482540	0.94
		6.9	483125	1.01
	**column temperature**(°C)	40	481667	1.16
		42	490602	0.85
		38	483003	0.98

a RSD % = [SD/mean] × 100.

All %RSD values remained below 2%, indicating that no significant deviations occurred in the analytical response. These findings confirm that the proposed method is robust and provides a consistent quantitative performance under slight, deliberate changes in chromatographic conditions. This stability highlights the methods’ suitability for routine implementation where small variations in environmental or instrumental conditions are inevitable. The robustness performance also supports their practical classification as “routine quality control-ready” methods within the BAGI framework.

### 3.3 Whiteness Assessment

The performance and applicability scores of the developed methods are given in Figure 4. The developed methods achieved total RAPI scores of 72.5 and 65 for HPLC-PDA and HPLC-ELSD, respectively, reflecting a moderate analytical performance. The BAGI scores demonstrate that the PDA-based approach offers superior practical performance. According to the CACI assessment, the developed HPLC-PDA and HPLC-ELSD methods for LMV determination achieved scores of 78 and 73, respectively, indicating that both approaches exhibit satisfactory practical performance.

**Figure 4:**
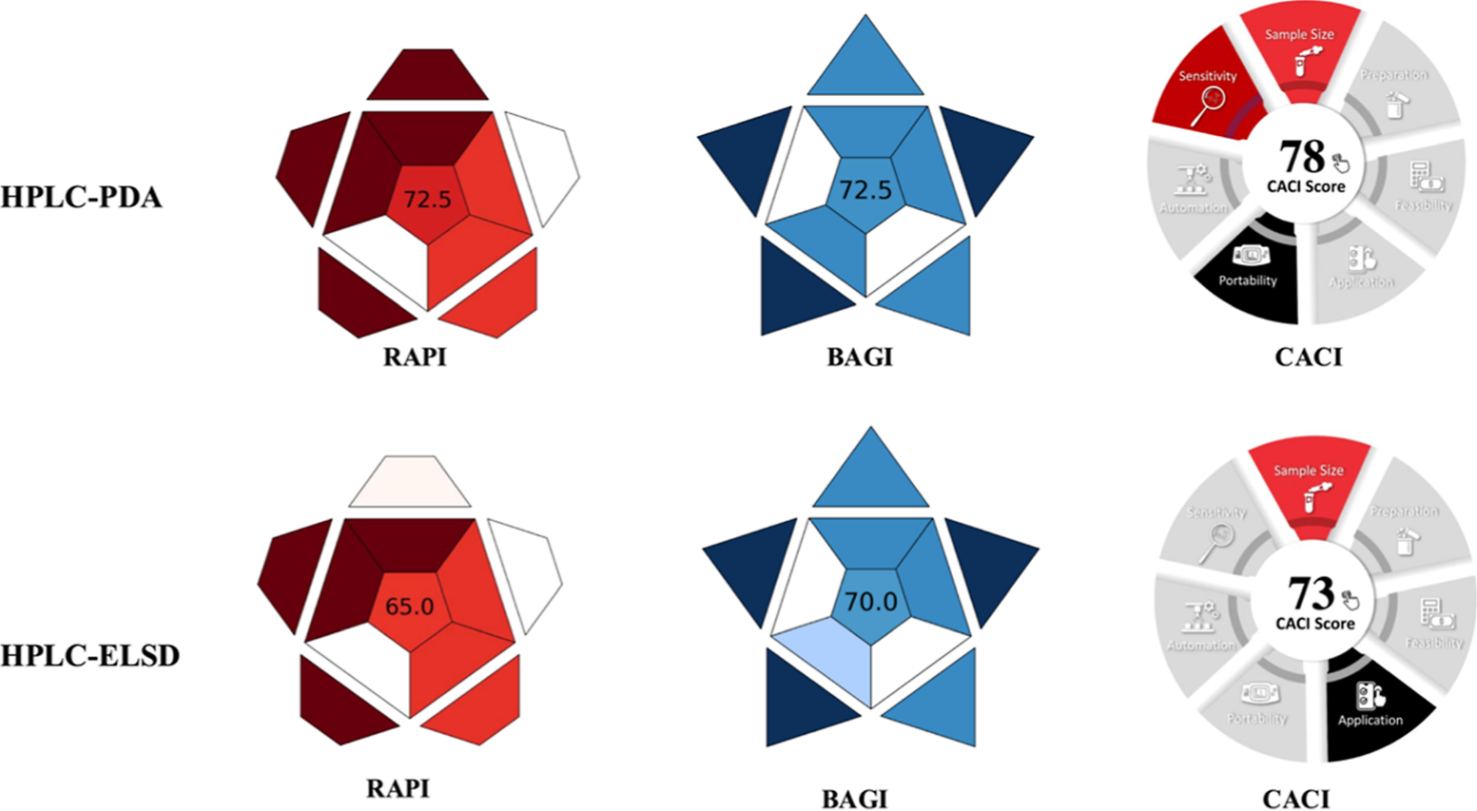
Performance and applicability scores of the developed methods.

Based on the AES evaluation, the developed HPLC-PDA and HPLC-ELSD methods for LMV determination achieved AES scores of 87 and 86, respectively, indicating that both approaches exhibit good environmental performance, with the HPLC-PDA method demonstrating superior greenness. The AES details of the developed methods are presented in Table 6.

**Table 6:** AES Scores for HPLC-PDA and HPLC-ELSD Methods

	HPLC-PDA	HPLC-ELSD
EtOH	4	4
water	0	0
HPLC	1	2
sonicator	0	0
balance	0	0
occupational hazard	0	0
waste	8	8
**total PPs**	**∑ 13**	**∑ 14**
**AES score**	**100–∑** = **87**	**100–∑** = **86**

The AGREE scores of the developed HPLC-PDA and HPLC-ELSD methods for LMV determination demonstrate satisfactory environmental performance, with the PDA-based method exhibiting superior greenness. In this study, both the HPLC-PDA and HPLC-ELSD methods yielded identical AGREEprep and SPMS scores for the sample preparation stage, corresponding to a moderate level of environmental sustainability, indicating a satisfactory compromise between analytical performance and environmental sustainability, while highlighting the potential areas for further improvement in the greenness of sample preparation. The AGSA and MoGAPI scores of the methods support the green claims of the proposed methods. The greenness metric evaluation of the methods is illustrated in Figure 5.

**Figure 5:**
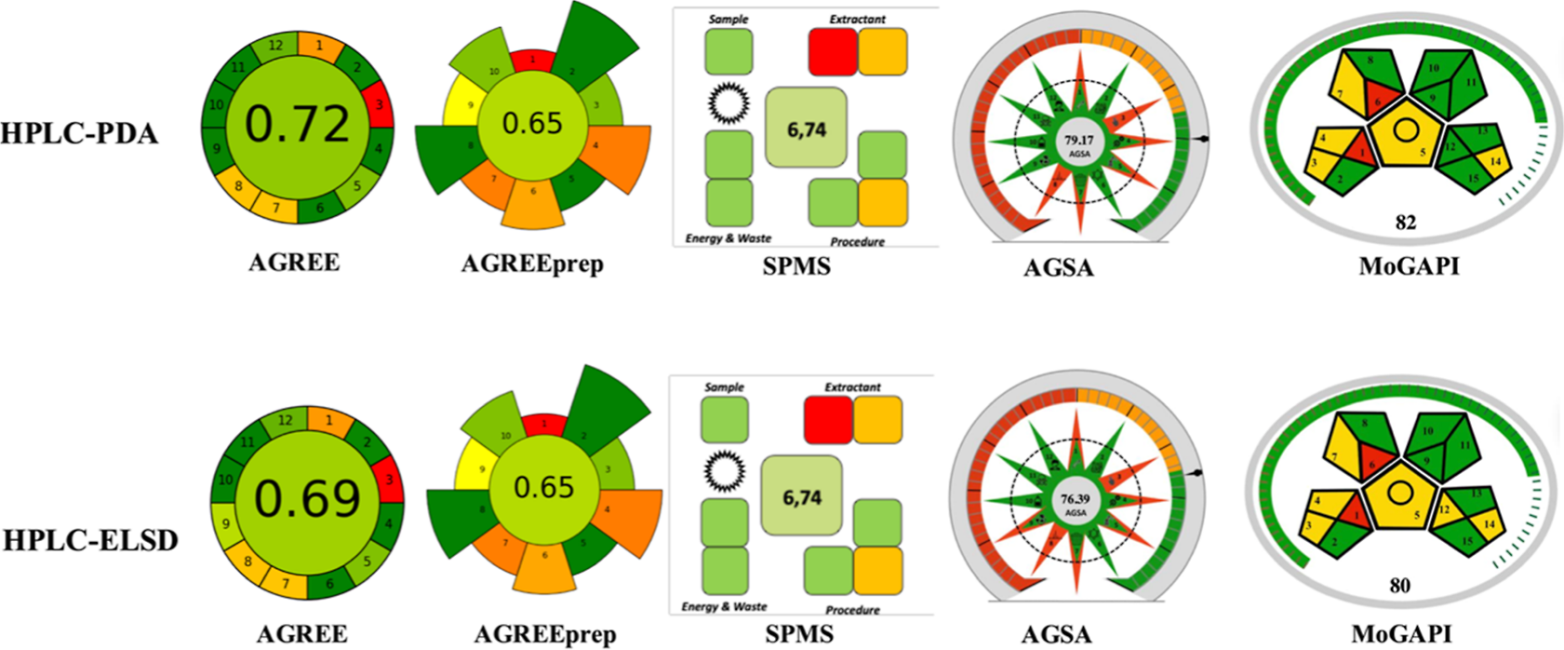
Greenness scores of the developed methods.

In addition to the applied greenness and applicability metrics, a deterministic evaluation of organic solvent consumption and electrical energy demand was considered to support the economic sustainability dimension of WAC. Under the applied chromatographic conditions (1.0 mL min^−1^ flow rate, 10 min run time, and 7% *v/v* EtOH), the organic solvent consumption can be deterministically calculated as approximately 0.7 mL of EtOH per analysis. Furthermore, the short and fixed analysis time combined with isocratic operation ensures a predictable and limited electrical energy demand per run. Although the ELSD detector requires additional energy for nebulization and evaporation, this consumption remains constant under fixed operating parameters, allowing controlled and reproducible energy usage per analysis.

From a sustainability perspective, the exclusive use of EtOH and water as mobile-phase components represented a significant advancement toward greener analytical practices. EtOH is recognized as a GRAS solvent, and its use minimized toxicity, waste generation, and environmental burden compared with ACN or MeOH typically used in HPLC assays. The very low organic solvent requirement (0.7 mL per run) further enhanced the ecological profile of the method.

Although the HPLC-ELSD method exhibited slightly lower overall whiteness compared with the PDA-based approach, this difference primarily stems from its higher LOQ and greater energy demand associated with the operation of the nebulization and evaporation units. These factors marginally affect its analytical efficiency and environmental score within the WAC framework. Nevertheless, even under these conditions, the proposed ELSD method demonstrated an acceptable analytical performance, satisfactory applicability, and an environmentally responsible profile. Therefore, both the developed methods can be considered sustainable, reliable, and well-balanced analytical alternatives that align with the holistic WAC concept by integrating accuracy, practicality, and ecological consciousness.

From an analytical decision-making perspective, the choice between PDA and ELSD detection should be guided by the intended application rather than by a single performance metric. The PDA method is preferable when high sensitivity, low limits of quantification, and trace-level determination are required, such as in low-dose formulations or quality control scenarios that demand stringent sensitivity. In contrast, ELSD offers a more universal detection approach and may be advantageous in cases involving UV-weak analytes, complex matrices, or laboratories seeking detector flexibility with acceptable sensitivity trade-offs.

To contextualize the performance of the proposed methods, a comparative assessment with selected literature methods was conducted (Table 7). Rather than providing an exhaustive review, two representative methods were chosen to illustrate typical analytical approaches reported for the LMV determination. This comparison highlights that the proposed methods achieve comparable analytical performance while offering advantages in terms of reduced organic solvent consumption and improved alignment with WAC principles.

**Table 7:**
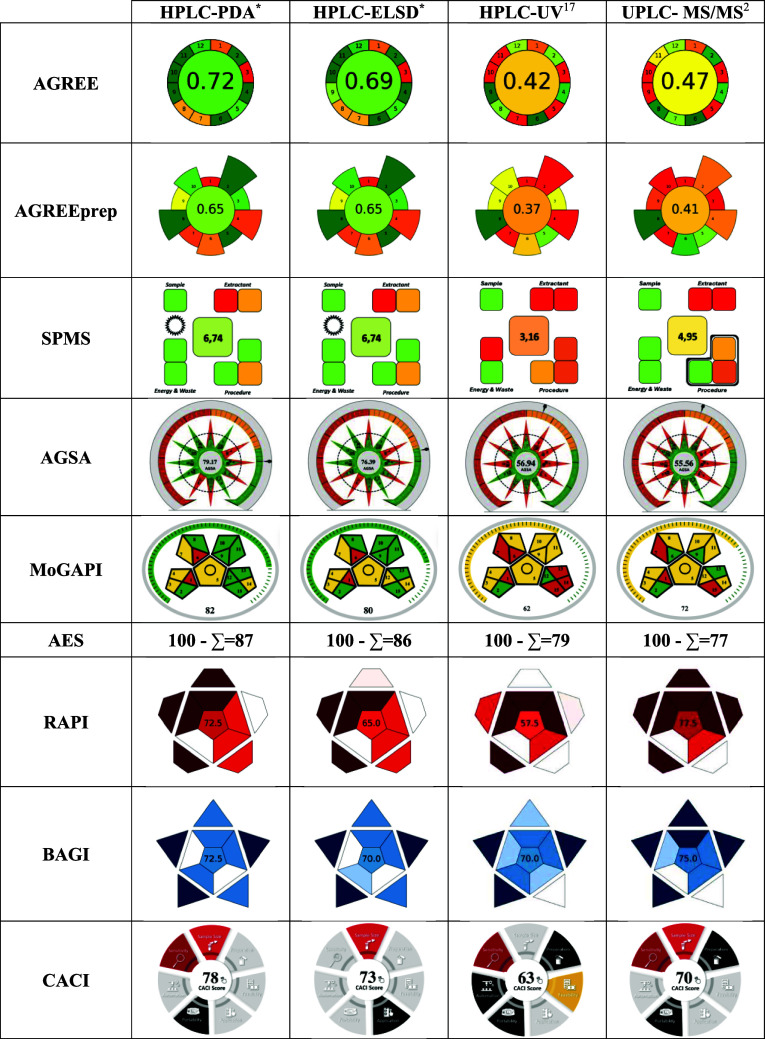
Comparative Evaluation of the Proposed and the Selected Literature Methods for LMV Determination^a^

a ^*^Proposed method.

### 3.4 Assay of Samples

A commercially available pharmaceutical formulation labeled to contain 100 mg of LMV was obtained and analyzed by using the developed HPLC-PDA and HPLC-ELSD methods to verify its content. The measured LMV amounts were 101.2 ± 0.68 and 100.8 ± 0.43, respectively, indicating acceptable consistency with the declared value and demonstrating the reliability of both analytical approaches. Although only a single commercial formulation was analyzed in this study, the robustness results and specificity evaluation suggest that the method is reliable. Future studies may include multiple commercial brands to further investigate the potential variability arising from different excipient compositions.

## 4 Conclusions

The developed HPLC-PDA and HPLC-ELSD methods provide accurate, precise, and reproducible quantification of LMV while adhering to the principles of sustainability. Using only 0.7 mL of EtOH as the organic solvent per analysis, the methods significantly minimize chemical waste and the ecological impact. The exclusive use of EtOH, a renewable and low-toxicity solvent, further aligns the approach with the GAC principles. Integrating analytical performance (red), environmental sustainability (green), and practical applicability (blue), the proposed methods fit seamlessly within the WAC framework. Among them, the HPLC-PDA system achieved the most balanced “whiteness” profile, combining high analytical efficiency with environmental responsibility. The validated HPLC-PDA and HPLC-ELSD methods developed in this study demonstrate robust analytical performance for tablet formulations and may be considered technically adaptable to other matrices, including pediatric syrups or biological samples, provided that appropriate matrix-specific sample preparation and validation strategies are applied in future studies. Although LMV can be reliably quantified using PDA due to its UV absorbance, PDA detection is inherently limited to chromophore-containing species. Impurities, degradation products, or excipients with weak or no UV absorption may not be adequately monitored by PDA. In this context, ELSD provides a complementary, mass-sensitive, and chromophore-independent detection capability, enhancing the method’s applicability and offering additional assurance for future impurity profiling and stability studies. Overall, this study not only introduces the first HPLC-ELSD method for LMV determination but also demonstrates how multimetric evaluation can lead to chromatographic methods that are both analytically robust and environmentally conscious, offering a model for future sustainable analytical practices.

## References

[1] Saka C. (2009). High-performance
liquid chromatography methods to simultaneous determination of anti-retroviral
drugs in biological matrices. Crit. Rev. Anal.
Chem..

[2] Kanjarla N., Katta B. (2025). Simultaneous quantification
of doravirine, lamivudine, and tenofovir
disoproxil fumarate in human plasma by UPLC-MS/MS: method development
and validation. Turk. J. Pharm. Sci..

[3] Alsharif S. T., Almalki A. H., Ramzy S., Sultan Alqahtani A., Abduljabbar M. H., Algarni M. A., Serag A. (2024). Derivative
spectroscopy
and wavelet transform as green spectrophotometric methods for abacavir
and lamivudine measurement. Spectrochim. Acta
A Mol. Biomol. Spectrosc..

[4] Kepekci
Tekkeli S. E. (2013). Extractive spectrophotometric method for the determination
of lamivudine and zidovudine in pharmaceutical preparations using
bromocresol purple. J. Chem..

[5] Mallepelli S., Kamera S., Garlapati A. (2022). Simultaneous estimation of lamivudine,
didanosine and efavirenz in bulk and their formulation by UPLC. Int. J. Pharm. Investig..

[6] Short W. R., Patel P., Verdier G., Puga A., Vannappagari V., de Ruiter A., Jones B. (2025). Role of dolutegravir/lamivudine in
the management of pregnant people living with HIV-1: a narrative review. Infect. Dis. Ther..

[7] Liu Y., Peng J., Liang Y., Li Y., Zhen X., Li H. (2025). QuEChERS and UPLC-MS/MS-based quantification of human plasma of eight
nucleoside reverse transcriptase inhibitors and platinum anticancer
drugs for hepatocellular carcinoma. Molecules.

[8] Deepali G., Elvis M. (2010). UV spectrophotometric
method for assay of the anti-retroviral agent
lamivudine in active pharmaceutical ingredient and in its tablet formulation. J. Young Pharm..

[9] Aboul-Enein H. Y., Hefnawy M. M. (2003). High throughput
analysis of lamivudine in pharmaceutical
preparations using monolithic silica HPLC column. Anal. Lett..

[10] Ozkan S. A., Uslu B. (2002). Rapid HPLC assay for
lamivudine in pharmaceuticals and human serum. J. Liq. Chromatogr. Relat. Technol..

[11] Somkuwar K., Sabale P., Sawale V., Rahangdale P. (2024). Comparative
study of UV spectroscopy, RP-HPLC and HPTLC methods for quantification
of antiviral drug lamivudine in tablet formulation. Futur. J. Pharm. Sci..

[12] Leandro K. C., Moreira J. C., Farias P. A. M. (2013). Differential
pulse voltammetric studies
on lamivudine: an antiretroviral drug. Am. J.
Analyt. Chem..

[13] Kapoor N., Khandavilli S., Panchagnula R. (2006). Simultaneous determination of lamivudine
and stavudine in antiretroviral fixed dose combinations by first derivative
spectrophotometry and high performance liquid chromatography. J. Pharm. Biomed. Anal..

[14] DrugBank Online . 2025. Lamivudine (DB00709). Retrieved from https://go.drugbank.com/drugs/DB00709(2025 11 04).

[15] Kenney K. B., Wring S. A., Carr R. M., Wells G. N., Dunn J. A. (2000). Simultaneous
determination of zidovudine and lamivudine in human serum using HPLC
with tandem mass spectrometry. J. Pharm. Biomed.
Anal..

[16] Chen X., Bu F., Li R., Yuan G., Wang Y., Wang B. (2019). Overview of
the chromatographic and mass spectrometry analytical methods for determination
of lamivudine in biological fluids. Curr. Pharm.
Anal..

[17] Kumar D. A., Rao G. S., Rao J. V. L. N. S. (2010). Simultaneous determination of lamivudine,
zidovudine and abacavir in tablet dosage forms by RP HPLC method. J. Chem..

[18] Kumar A., Majee C., Namdev V. (2020). Analytical
method development and
validation of lamivudine in formulation by using reversed phase ultra
performance liquid chromatography. Int. J. PharmTech
Res..

[19] Subrahmanyam E. V. S., Kumara Prasad S. A., Shabaraya A. R. (2018). Validated
spectroscopic methods for the determination of fluoxetine HCl and
lamivudine in bulk and marketed formulations. Int. J. Health Sci. Pharm..

[20] Thobakgale L., Thwala L. N., Mthunzi-Kufa P. (2025). Detection
of lamivudine using liquid-surface-enhanced
Raman spectroscopy. RSC Adv..

[21] Sekar R., Azhaguvel S. (2008). MEKC determination of antiretroviral reverse transcriptase
inhibitors lamivudine, stavudine, and nevirapine in pharmaceutical
formulations. Chromatographia.

[22] Hussain C. M., Hussain G., Keçili R. (2025). Disposable
analytical chemistry:
design, application & sustainability. TrAC
- Trends Analyt. Chem..

[23] Hussain C. M., Hussain G., Keçili R. (2025). Smart analytical
chemistry: Integrating
green, sustainable, white and AI-driven approaches in modern analysis. TrAC - Trends Analyt. Chem..

[24] Hussain C. M., Hussain G., Ünlüer O. ¨., Keçili R. (2025). White analytical chemistry-driven
electroanalytical
strategies for sustainable detection of pharmaceuticals: Design and
applications. Electrochim. Acta.

[25] Sezgin B., Arli G., Soyseven M. (2025). Multi-metric
greenness, performance
and applicability evaluation of a green HPLC-ELSD method for polysorbate
80 determination in various pharmaceuticals compared with AI-assisted
scoring systems. Microchem. J..

[26] Sezgin B., Soyseven M., Arli G. (2024). Greenness assessment
and comparison
of the developed and validated green HPLC-PDA, HPLC-FLD, and HPLC-ELSD
methods for the determination of melatonin in various products using
analytical eco-scale, NEMI, GAPI, and AGREE greenness metric tools. Microchem. J..

[27] Committee for Medicinal Products for Human Use . ICH Harmonised Guideline: Validation of analytical procedures-Q2 (R2). ICH Eur. Med. Agency 2022.

[28] Nowak P. M., Wojnowski W., Manousi N., Samanidou V., Płotka-Wasylka J. (2025). Red analytical performance index (RAPI) and software:
the missing tool for assessing methods in terms of analytical performance. Green Chem..

[29] Manousi N., Wojnowski W., Płotka-Wasylka J., Samanidou V. (2023). Blue applicability
grade index (BAGI) and software: a new tool for the evaluation of
method practicality. Green Chem..

[30] Mansour F. R., Bedair A., Locatelli M. (2025). Click Analytical
Chemistry Index
as a novel concept and framework, supported with open source software
to assess analytical methods. Adv. Sample Prep..

[31] Larsen C., Lundberg P., Tang S., Ràfols-Ribé J., Sandström A., Mattias Lindh E., Wang J., Edman L. (2021). A tool for
identifying green solvents for printed electronics. Nat. Commun..

[32] Gałuszka A., Migaszewski Z. M., Konieczka P., Namieśnik J. (2012). Analytical
Eco-Scale for assessing the greenness of analytical procedures. TrAC - Trends Analyt. Chem..

[33] Pena-Pereira F., Wojnowski W., Tobiszewski M. (2020). AGREE - Analytical
GREEnness metric
approach and software. Anal. Chem..

[34] Wojnowski W., Tobiszewski M., Pena-Pereira F., Psillakis E. (2022). AGREEprep
Analytical greenness metric for sample preparation. TrAC - Trends Analyt. Chem..

[35] González-Martín R., Gutiérrez-Serpa A., Pino V., Sajid M. (2023). A tool to
assess analytical sample preparation procedures: Sample preparation
metric of sustainability. J. Chromatogr. A.

[36] Mansour F. R., Płotka-Wasylka J., Locatelli M. (2024). Modified GAPI
(MoGAPI) tool and software
for the assessment of method greenness: case studies and applications. Analytica.

